# Comparison of software packages for detecting differential expression in RNA-seq studies

**DOI:** 10.1093/bib/bbt086

**Published:** 2013-12-02

**Authors:** Fatemeh Seyednasrollah, Asta Laiho, Laura L. Elo

**Keywords:** RNA-seq, gene expression, differential expression

## Abstract

RNA-sequencing (RNA-seq) has rapidly become a popular tool to characterize transcriptomes. A fundamental research problem in many RNA-seq studies is the identification of reliable molecular markers that show differential expression between distinct sample groups. Together with the growing popularity of RNA-seq, a number of data analysis methods and pipelines have already been developed for this task. Currently, however, there is no clear consensus about the best practices yet, which makes the choice of an appropriate method a daunting task especially for a basic user without a strong statistical or computational background. To assist the choice, we perform here a systematic comparison of eight widely used software packages and pipelines for detecting differential expression between sample groups in a practical research setting and provide general guidelines for choosing a robust pipeline. In general, our results demonstrate how the data analysis tool utilized can markedly affect the outcome of the data analysis, highlighting the importance of this choice.

## INTRODUCTION

Deep sequencing of RNAs (RNA-seq) has rapidly become the tool of choice in many genome-wide transcriptomic studies. It enables the annotation and quantification of genes across various samples with improved sensitivity over expression microarrays [1–3]. A fundamental research problem in many RNA-seq studies is the identification of differentially expressed genes between distinct sample groups (e.g. healthy and disease). Together with the growing popularity of the RNA-seq technology, a number of computational software packages and pipelines have already been introduced for this task, including methods based on negative binomial models such as edgeR [4], DESeq [5] and baySeq [6], non-parametric approaches such as NOIseq [7] and SAMseq [8], transformations of gene-level read counts for linear modeling with limma [9], as well as transcript-based detection methods that also enable gene level differential expression reports, such as Cuffdiff 2 [10] and EBSeq [11]. Currently, however, the understanding of the best practices remains rather shallow and the field is under continuous development [1, 12].

In general, it seems that similar challenges and solutions as earlier with microarrays are now debated in the context of RNA-seq. For instance, the early microarray studies involved often only very few replicate samples and they determined differential expression using simplistic statistics, such as fold-change, while it soon became evident that it is essential to consider also the variability over replicate samples. Therefore, microarray experiments today make use of advanced statistical testing procedures, such as those based on modified *t*-tests [9]. Similarly, the early RNA-seq studies often used a single source of RNA and the counts across technical replicates were reported to fit well to a Poisson distribution [3, 12, 13]. However, when data on biological replicates became more available, it was soon discovered that the variability between replicated measurements is often higher than expected by Poisson distribution—a phenomenon called overdispersion—making Poisson-based analyses prone to high false positive rates. Consequently, methods to deal with biological variability and overdispersion have been introduced, such as methods based on negative binomial and beta negative binomial models [4–6, 14]. As the detection of differentially expressed genes involves performing a large number of statistical tests, multiplicity needs to be taken into account when determining the significance of the detections. The conventional correction of *P* values for multiplicity aims to control the family-wise error rate, but it is often too conservative for the purposes of many biological studies. Therefore, when assessing the statistical significance of the detections, it has become a common practice to control the false discovery rate (FDR) of the detections, that is, the expected proportion of false positives among all the detections, and correct the *P* values accordingly using, for instance, the Benjamini–Hochberg method [15].

Although several recent studies have introduced new software packages to detect differential expression from RNA-seq data sets and the authors have shown their improved performance over previous methods in selected data sets, only few independent comparisons have been published so far. In particular, a systematic independent comparison of the state-of-the-art methods and pipelines in real data sets at different numbers of replicates is still lacking. Toward an independent comparison, Kvam *et al.* [12] compared recently the performance of four related packages (edgeR, DESeq, baySeq and TSPM) but focused mainly on simulated data sets. Soneson and Delorenzi [16] included in their comparison a total of 11 approaches (edgeR, DESeq, baySeq, NBPSeq, TSPM, EBSeq, NOIseq, SAMseq, ShrinkSeq and two versions of limma) but again focused mainly on simulated data sets. Nookaew *et al.* [17] included in their comparison five packages (edgeR, DESeq, baySeq, NOIseq and Cuffdiff) on a real data set but their case study on *Saccharomyces cerevisiae* involved only three replicates per group. None of these comparisons included the very recently introduced Cuffdiff 2, which has been proposed to outperform the previous approaches [10].

To this end, we present here a systematic practical pipeline comparison of eight software packages edgeR, DESeq, baySeq, NOIseq, SAMseq, limma, Cuffdiff 2 and EBSeq, which represent the current state-of-the-art of the field. All of them except Cuffdiff 2 are available in R or Bioconductor. To complement the previously published simulation studies under a more realistic setting, we focus here on two publicly available real data sets that are among the few with relatively large numbers of replicates. These data allow us to evaluate the different software packages and pipelines at different numbers of replicates covering a variety of practical research settings. The use of publicly available data sets also guarantees that any future analysis package can be easily compared against the results. As our goal is to provide an objective and practical assessment of the packages in a sense of real experimental pipelines and guide an average user in choosing a robust data analysis pipeline, we apply each of the packages following the instructions provided in the software manuals—as most users in practice typically do. For evaluation, we focus on measures that are of practical interest to researchers when analyzing their data sets. In particular, we investigate the number of detections at different numbers of replicates, their consistency within and between pipelines, the estimated proportion of false discoveries and the runtimes. In general, we illustrate that there are marked differences between the different software packages, which can lead to considerable variability in reported results. To aid the selection of a suitable data analysis approach, we provide some practical guidelines taking into consideration the experimental design and goals of the analysis (e.g. number of samples available, acceptable rates of false positives).

## METHODS

### Software packages for detecting differential expression

We give here a brief summary of the software packages that we compare in the present work for detecting differential expression between two groups of samples. For more detailed description of the packages and the statistical models they apply, the reader is referred to the original publications and the related software package websites (Table 1). When applying the packages, we focus on the approach that an average user is likely to take, that is, we follow the instructions and recommendations provided in the software manuals about the implementation and parameters, including the default normalization methods. Additionally, we also consider the use of similarly preprocessed data in the analyses using the Trimmed Mean of M values (TMM) normalization [18], which was recently recommended in an extensive comparison of various normalization methods [19]. This enables us to assess the overall effect of normalization on the outcome of the different packages and more directly assess the differences between the statistical tests of the packages. The codes used to perform the calculations are available as Supplementary Code.

**Table 1:** Software packages for detecting differential expression

Method	Version	Reference	Normalization^a^	Read count distribution assumption	Differential expression test
edgeR	3.0.8	[4]	TMM/Upper quartile/RLE (DESeq-like)/None (all scaling factors are set to be one)	Negative binomial distribution	Exact test
DESeq	1.10.1	[5]	DESeq sizeFactors	Negative binomial distribution	Exact test
baySeq	1.12.0	[6]	Scaling factors (quantile/TMM/total)	Negative binomial distribution	Assesses the posterior probabilities of models for differentially and non-differentially expressed genes via empirical Bayesian methods and then compares these posterior likelihoods
NOIseq	1.1.4	[7]	RPKM/TMM/Upper quartile	Nonparametric method	Contrasts fold changes and absolute differences within a condition to determine the null distribution and then compares the observed differences to this null
SAMseq (samr)	2.0	[8]	SAMseq specialized method based on the mean read count over the null features of the data set	Nonparametric method	Wilcoxon rank statistic and a resampling strategy
Limma	3.14.4	[9]	TMM	voom transformation of counts	Empirical Bayes method
Cuffdiff 2 (Cufflinks)	2.0.2-beta	[10]	Geometric (DESeq-like)/quartile/classic-fpkm	Beta negative binomial distribution	*t*-test
EBSeq	1.1.7	[11]	DESeq median normalization	Negative binomial distribution	Evaluates the posterior probability of differentially and non-differentially expressed entities (genes or isoforms) via empirical Bayesian methods

^a^In case of availability of several normalization methods, the default one is underlined.

**edgeR** [4] determines differential expression using empirical Bayes estimation and exact tests based on a negative binomial model. The package has been developed to enable analysis of experiments with small numbers of replicates. In particular, an empirical Bayes procedure is used to moderate the degree of overdispersion across genes by borrowing information between genes. An exact test analogous to Fisher’s exact test but adapted to overdispersed data is used to assess differential expression for each gene. As default, the TMM normalization procedure is carried out to account for the different sequencing depths between the samples, whereas the Benjamini–Hochberg procedure is used to control the FDR [15].

**DESeq** [5] uses a similar negative binomial model as edgeR but models the observed relationship between the mean and variance when estimating dispersion, allowing a more general, data-driven parameter estimation. According to the method developers, this allows a balanced selection of differentially expressed genes throughout the dynamic range of the data. Similar to edgeR, a scaling factor normalization procedure is carried out to account for the varying sequencing depths of the different samples and the Benjamini–Hochberg procedure is used to control the FDR. Also DESeq has been developed to enable analysis of experiments with small numbers of replicates. With DESeq, it is technically possible, although not recommended, to work with experiments without any biological replicates.

**baySeq** [6] is based on estimating posterior likelihoods of differential expression via empirical Bayesian methods, assuming negative binomially distributed data. The method produces posterior probabilities rather than significance values and reports a Bayesian FDR estimate. The varying library sizes are taken into account by library scaling factors. The method is relatively computationally intensive but, unlike the other R/Bioconductor packages, its implementation makes it directly possible to take advantage of parallel processing (not applied in this study).

**NOIseq** [7] is a data adaptive non-parametric method, which empirically models the noise distribution from the actual data by contrasting fold-change differences and absolute expression differences among samples within the same condition. According to the method developers, it can therefore adapt to the size of the data set and thus efficiently control the rate of false discoveries. NOIseq includes RPKM (reads per kilobase per million mapped reads) [2] as a default normalization method. The package is specifically designed to account for small numbers of replicates and genes with low expression levels.

**SAMseq** [8] is a non-parametric method based on Wilcoxon rank statistic and a resampling procedure to account for the different sequencing depths. A permutation-based approach is used to estimate FDR. According to the method developers, it can be applied to data with at least moderate numbers of replicate samples (∼10 or more). It is claimed that the method is able to select significant features more efficiently than parametric methods in cases when their distributional assumptions do not hold.

**Limma** [9] is based on linear modeling. It was originally designed for analyzing microarray data but has recently been extended to RNA-seq data. The current recommendation according to the limma user guide is to use TMM normalization of the edgeR package and the so called ‘voom’-conversion which essentially transforms the normalized counts to logarithmic (base 2) scale and estimates their mean–variance relationship to determine a weight to each observation prior to linear modeling [20]. By default, the Benjamini–Hochberg procedure is used to estimate the FDR [15].

**Cuffdiff 2** [10] estimates expression at transcript-level resolution and controls for variability and read mapping ambiguity by using a beta negative binomial model for fragment counts. It is part of the extensive Cufflinks package developed for the identification of differentially expressed genes and transcripts and revealing differential splicing and promoter-preference changes. Although Cuffdiff 2 enables to analyze signals at the transcript level, it reports differential expression also at the gene level and these gene level results were used here as a basis for comparison with the other software packages. By default, Cuffdiff 2 uses a similar scaling factor procedure as DESeq to account for the different sequencing depths and the Benjamini–Hochberg procedure to control the FDR. The Cuffdiff 2 method specifically addresses the uncertainties in counts owing to ambiguous reads that easily result in false differential expression calls of genes especially with several similar isoforms. In this work, we only looked at the gene level analysis results of the pipeline.

**EBSeq** [11] is mainly developed to identify differentially expressed isoforms but has demonstrated a robust outcome in gene level analyses as well. EBSeq estimates the posterior likelihoods of differential and equal expression by the aid of empirical Bayesian methods, assuming negative binomial distribution for the data. To account for the different sequencing depths, a median normalization procedure similar to DESeq is used as the default method [5]. A Bayesian FDR estimate is provided.

To use comparable thresholds for determining the differentially expressed genes with the different packages, we used the FDR of 0.05 for all packages except NOIseq, which does not report any FDR estimate. To obtain as comparable threshold as possible with NOIseq, we required that the probability of differential expression was above 0.8, as suggested by the method developers. When determining the ranking of the genes, we primarily ranked the genes according to their multiple testing corrected significance levels reported by the software packages and secondarily according to the nominal significance levels as the corrected values in many cases quickly reached their maximum value. These ranks were used for determining the similarity between the different methods by calculating the Spearman correlation of the gene ranks.

Along with development of this study, a new version of the DESeq package named DESeq2 (v.1.1.25) and a new version of Cuffdiff 2 named Cuffdiff 2.1 (v.2.1.1) were released but the corresponding manuscripts have not been published yet. Hence, although we included DESeq2 and Cuffdiff 2.1 in our comparison analyses as well, we did not incorporate the results in the main part of our work. However, to provide as complete as possible information to interested readers, we present a summary of the results in Supplementary Figures S1 and S2. In general, DESeq2 and Cuffdiff 2.1 showed not only an increase in the number of detections, when compared with DESeq and Cuffdiff 2, respectively, but also an increased number of false positives.

### Data sets

For a practical comparison of the software packages and pipelines in real data sets, we considered two publicly available data sets that were among the few with relatively large numbers of replicates. Additionally, these data represented diverse types of experiments, including mouse and human data with different levels of heterogeneity between replicate samples. In particular, the mouse data showed significantly higher correlations among replicate samples than the human data (Wilcoxon test, *P* < 0.01), suggesting that they constituted more homogeneous sample groups (Supplementary Figure S3).

The mouse RNA-seq data has been published in [21]. It consists of the striatum samples of 21 mice, 10 of the C57BL/6J strain and 11 of the DBA/2J strain. The samples were sequenced on Illumina Genome Analyzer II with 76 bp read length, yielding on average 22 million raw reads per sample. For the evaluation, we identified differentially expressed genes between the two strains, similarly as in the original publication [21].

The human RNA-seq data is part of the International HapMap project and it has been published in [22]. We considered here samples from lymphoblastoid cell lines of 56 unrelated Nigerian individuals, 28 males and 28 females. The samples were sequenced on Illumina Genome Analyzer II instrument with 35 and 46-bp read length. For the evaluation, we identified differentially expressed genes between males and females.

To include Cuffdiff 2 into our comparisons, we realigned all the sequenced read data using the latest reference sequences and annotations. The human data were aligned against the hg19 reference and the mouse data against the mm10 reference genome using the Tophat aligner with default parameters [14]. The average alignment rate was 0.89 (s.d. 0.02) and 0.86 (s.d. 0.03) for human and mouse, respectively. For all other methods except Cuffdiff 2, the gene counts were extracted using the HTSeq python tool (http://www-huber.embl.de/users/anders/HTSeq). In all cases, the genes were analysed against RefSeq gene annotations.

The Bioconductor package easyRNASeq [23] was used to calculate the RPKM values [2], which were utilized to divide the genes into four categories: very lowly or not expressed, lowly, medium or highly expressed genes. Briefly, following the approach of Hackett *et al.* [24], genes with an average RPKM value across samples below 0.125 were considered as very lowly or not expressed, genes with an average RPKM value between 0.125 and 1 were considered lowly expressed, between 1 and 10 medium expressed, and above 10 highly expressed.

To estimate the proportion of false discoveries, we utilized the replicates within the sample groups in the mouse and human data. More specifically, we constructed artificial two-group comparisons with different numbers of replicates by randomly sampling without replacement two subsets of samples from a single sample group 10 times for each sample group. We expected that no significant detections should be made in such mock comparisons. To compare between the different software packages, we divided the number of mock detections with the average number of detections in the actual comparisons with the same number of replicates. Only statistically significant genes were considered with each package.

## RESULTS AND DISCUSSION

### Effect of normalization on the detections

**Figure 1:**
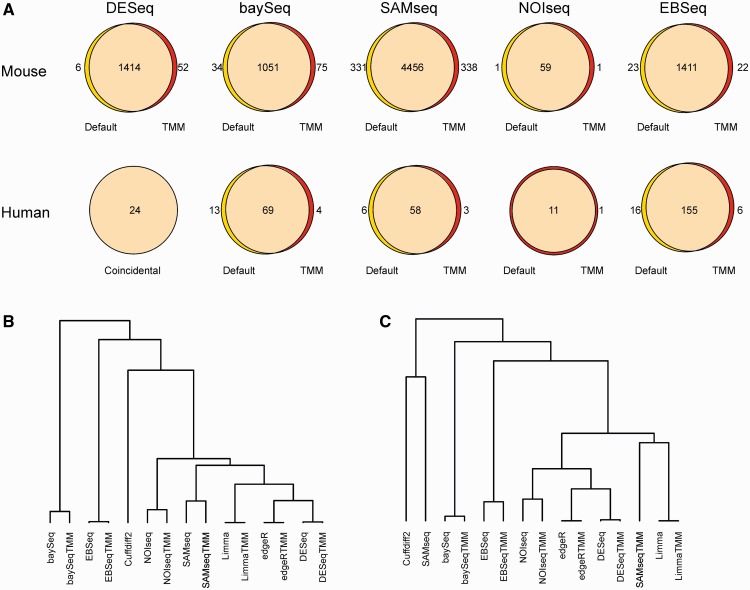
Effect of normalization on the detections. (**A**) Overlaps of the differentially expressed genes detected in the mouse and human data using the default or TMM normalization method for all R-based packages that do not use TMM as their default normalization option (see Methods for details of the significance thresholds). With edgeR and limma the TMM normalization is the default normalization and, therefore, they are not included. Overall similarity between the rankings obtained using the default or TMM normalization method (denoted by TMM after the name of the package) in the (**B**) mouse and (**C**) human data. The dendrogram was constructed using average linkage hierarchical clustering and Spearman correlation of the gene ranks.

To assess the effect of normalization on the outcome of the different packages, we considered both the use of the default normalization of each package, as well as the use of similarly preprocessed data normalized with the TMM normalization [18]. In particular, we investigated the differentially expressed genes detected in the mouse and human data when the number of replicates was varied. For this, subsamples of different sizes were generated from the complete data by group-preserving random sampling without replacement 10 times at each number of replicates and data. When looking at the number of detections at different numbers of replicates, no systematic significant differences were observed between the default and TMM normalization-based detections (Wilcoxon test, *P* > 0.1; Supplementary Figure S4). A closer look at the detections revealed a high overlap; with each software package and data, over 80% of the detections made using either of the normalizations were made using both of the normalizations (Figure 1A). Finally, comparison of the gene rankings confirmed the overall similarity of the results (Figure 1B and C). With the exception of SAMseq in the human data, the default and TMM normalization-based rankings were always grouped together in the dendrograms; the similarity between two differential expression rankings was determined by Spearman correlation of the gene ranks. Thus, to our surprise, changing the default normalization methods to TMM normalization did not have a significant impact on the results in the present comparisons. Therefore, for the rest of the results, we focus on the default normalization methods that an average user is likely to use.

### Number and consistency of detections

**Figure 2:**
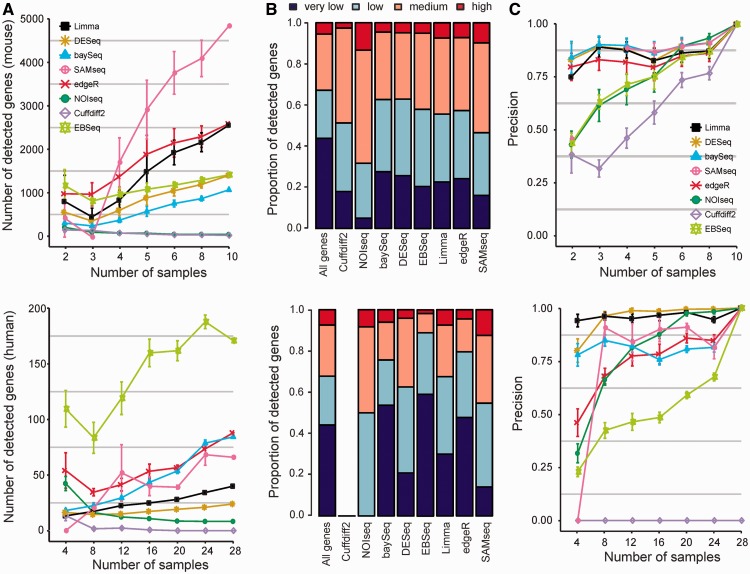
Number and consistency of differentially expressed genes detected using eight state-of-the-art software packages in the mouse and human data (upper and lower panel, respectively). (**A**) Number of detections (*y* axis) with different numbers of replicates (*x* axis) for each software package. The points correspond to averages over 10 randomly sampled subsets; the error bars show the standard error of the mean. (**B**) Differentially expressed genes in the complete data divided into four categories on the basis of their expression levels: very lowly or not expressed genes, lowly, medium and highly expressed genes. The different software packages were ordered on the basis of their total number of detections in the mouse data. (**C**) Precision of the detections (*y* axis) when increasing the number of replicates (*x* axis) in terms of genes identified as differentially expressed genes in the complete data using all the samples available in the mouse and human data (upper and lower panel, respectively). Only statistically significant genes were considered with each method (see Methods for details of the significance thresholds). The points correspond to averages over 10 randomly sampled subsets; the error bars show the standard error of the mean. A colour version of this figure is available at BIB online: http://bib.oxfordjournals.org.

As expected, with most of the packages, the number of detected differentially expressed genes increased when the number of replicate samples was increased (Figure 2A). However, with NOIseq and Cuffdiff 2, the number of detections decreased when the number of samples was increased. The same trend was observed both in the mouse and in the human data, suggesting that NOIseq and Cuffdiff 2 have low power especially when the number of replicates in the groups increases. For instance, Cuffdiff 2 did not identify any differentially expressed genes between the males and females in the complete human data. It was unable to detect even those genes on the male-specific chromosome Y that were identified as differentially expressed between males and females with all the other software packages. As NOIseq compares the changes between conditions to those within conditions aiming at low false positive rates, the method may not be ideally suited to such heterogeneous data sets as those in our comparisons. With Cuffdiff 2, the decreasing number of detections is likely related to the inability of the variability estimation to take into account the increasing number of replicates [10]. We also tested an older version of Cuffdiff (v.1.3.0) and different parameter settings but obtained similar results (Supplementary Table S1). With the recently released new version, however, an increase in the number of detections was observed but no supporting manuscript has been published yet (Supplementary Figure S1).

NOIseq and Cuffdiff 2 were generally most conservative, whereas the largest numbers of detections were typically obtained with edgeR and SAMseq, with the exception that SAMseq identified only few genes with small numbers of replicates when the non-parametric approach was also expected to have relatively low power. The number of detections with DESeq and limma was typically somewhere between the extremes, DESeq being more conservative than limma. The relative number of detections with baySeq and EBSeq varied depending on the data, being relatively low in the mouse data and among the highest in the human data. SAMseq showed the largest variability between the different subsets of the same size.

To take a closer look at the expression levels of the detected genes, we divided them into four categories on the basis of their average expression level across the samples: very low or not expressed, low, medium and high [24]. When considering all the genes in the data, the proportion of very lowly or not expressed genes was nearly 50% for both mouse and human (Figure 2B). Among the genes identified as differentially expressed, this proportion typically decreased below 35% but was still substantial. In particular, with edgeR, baySeq and EBSeq in the human data ∼50% or more of the detections were very lowly or not expressed. In general, our comparison illustrated that a large proportion of the detections involved relatively low count genes, requiring caution when interpreting the results. Although for the purposes of the present evaluation, we wanted to keep all the genes in the analyses, in practical research studies it may be reasonable to filter out the very lowly expressed genes from the results, especially with baySeq, edgeR and EBSeq but also with other methods. This can be done using, for instance, the overall sum of counts independent of the sample groups [16, 25] or the maximum normalized count across samples [26].

Finally, we examined the consistency of the detections between subsets of varying numbers of replicates and the complete data by calculating their overlap [27, 28]. More specifically, we determined the lists of differentially expressed genes in the subsets of different sizes with each software package and then calculated their average overlap with those identified as significant in the complete data (Figure 2C). The precision in terms of the complete data was generally highest with DESeq and limma. In the mouse data, also baySeq and SAMseq performed similarly when there were more than four replicates. The lowest precision was observed with NOIseq and Cuffdiff 2, which was in line with the observation that with them the number of detections decreased when increasing the number of replicates. Additionally, EBSeq showed low precision.

### False discoveries

**Figure 3:**
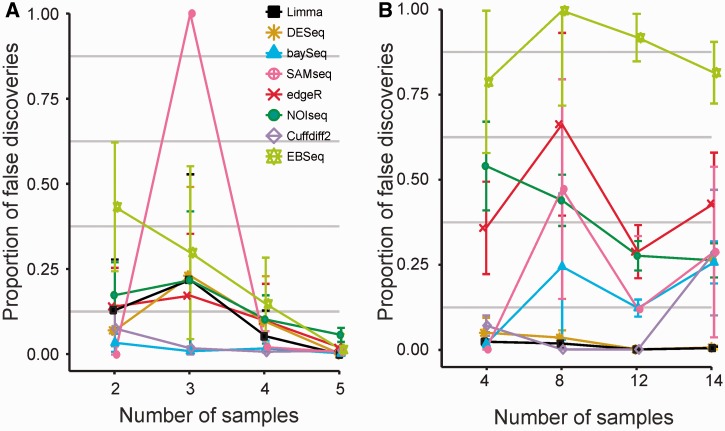
False discoveries on the basis of mock comparisons in the (**A**) mouse and (**B**) human data. In each mock comparison, differentially expressed genes were identified between two artificially constructed sample subsets from a single sample group, in which no significant detections are expected. To compare between the different software packages, we divided the number of mock detections with the average number of detections in the actual comparisons with the same number of replicates. Only statistically significant genes were considered with each method (see Methods for details of the significance thresholds). The points correspond to averages over 10 randomly sampled subsets; the error bars show the standard error of the mean. A colour version of this figure is available at BIB online: http://bib.oxfordjournals.org.

To assess how the different software packages and pipelines can control false positive rates, we utilized the multiple replicates within the sample groups by constructing artificial two-group comparisons. No significant detections were expected in such mock comparisons. Not unexpectedly, the proportion of false discoveries tended to decrease when increasing the number of replicates especially in the mouse data with relatively homogeneous sample groups (Figure 3 and Supplementary Tables S2 and S3**)**. In the mouse data, the differences between the software packages were relatively small, the most conservative baySeq and Cuffdiff 2 showing the lowest numbers of false detections and EBSeq in general the largest. SAMseq showed the largest variability. For instance, in the mouse data with only three replicates it sometimes detected a large number of genes also in the mock comparisons (Supplementary Table S2). In the human data, differences between the packages were larger and so was also the variability between the different subsets, reflecting the inherently larger heterogeneity between the samples. Now, DESeq, limma and Cuffdiff 2 showed the lowest proportions of false detections, whereas edgeR and EBSeq identified a relatively large number of genes as differentially expressed also in the mock comparisons. Notably, while NOIseq was relatively conservative in the actual comparisons, it identified relatively many genes in the mock comparisons.

### Similarity between methods

**Figure 4:**
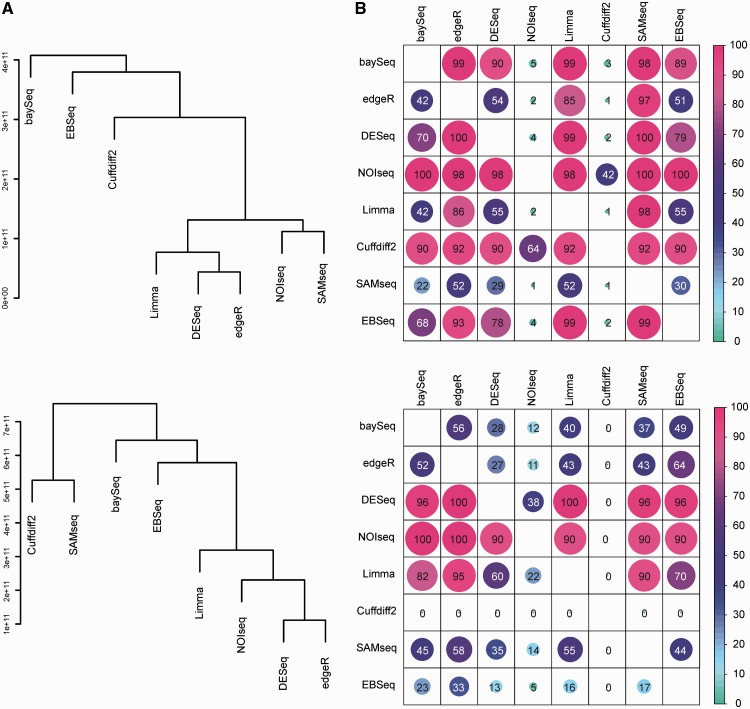
Similarity between the methods in the mouse and human data (upper and lower panel, respectively). (**A**) Overall similarity between the methods based on Spearman correlation of gene ranks. The dendrograms were constructed using average linkage hierarchical clustering. (**B**) Overlap of significant detections between the methods (see Methods for details of the significance thresholds). The proportion of common detections was calculated for each pair of methods, resulting in an asymmetric matrix of percentages. A colour version of this figure is available at BIB online: http://bib.oxfordjournals.org.

Finally, we assessed the similarities and differences between the different procedures. To characterize the overall similarities of the results, we first compared the obtained gene rankings using Spearman correlation (Figure 4A and Supplementary Figures S5 and S6). This suggested that the rankings obtained with baySeq, Cuffdiff 2 and EBSeq were generally most different from the rest of the software packages. On the other hand, the most similar ranking results were obtained with DESeq and edgeR, which share also the same underlying statistical model. The behavior of SAMseq seemed to be highly dependent on the data. The higher overall correlations between the gene rankings in the mouse data, as compared with those in the human data, could be attributed to larger differences between the mouse groups as well as higher homogeneity of the samples within the groups (Supplementary Figure S3).

Besides the overall rankings, we investigated also the overlaps of the significant detections between the software packages (Figure 4B). This showed that the genes identified with the more conservative pipelines were typically detected also with the less conservative ones. For instance, in the mouse data, the lowest number of genes was identified with Cuffdiff 2 (Figure 2A) and at least 90% of these genes were typically detected with the other packages (Figure 4B). In addition to NOIseq and Cuffdiff 2, a large percentage of the detections made with DESeq were identified with most of the other software packages. In contrast, SAMseq identified a relatively large number of genes that were typically not detected with the other packages.

### Runtimes

**Figure 5:**
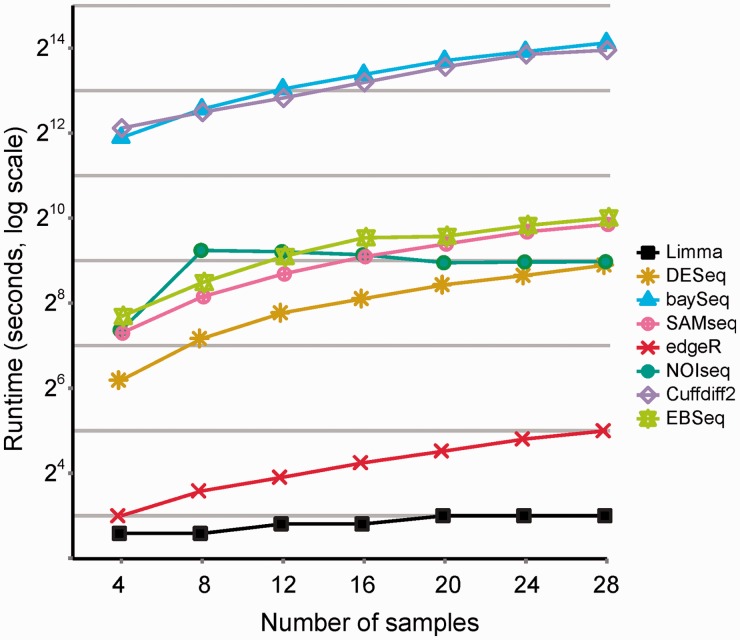
Runtimes of the different methods to identify differentially expressed genes in the human data. Time in seconds on log scale (*y* axis) is shown as a function of the number of replicates (*x* axis). The analyses were run on a computer cluster node with two Intel XEON Hexa-Core processors and 96 GB of memory. Cuffdiff 2 was run on all the available 12 cores; the R/Bioconductor packages were run on a single core. A colour version of this figure is available at BIB online: http://bib.oxfordjournals.org.

The computational time required to run the analyses varied a lot depending on the software package (Figure 5); edgeR and limma were computationally fastest taking only seconds to run, whereas baySeq and Cuffdiff 2 took hours to run. The computational time required by DESeq, SAMseq, NOIseq and EBSeq was in minutes, ranging from 1 to ∼15 minutes depending on the number of replicates. The analyses were run on a computer cluster node with two Intel XEON Hexa-Core processors and 96 GB of memory. The only software that directly supported multi-threading was Cuffdiff 2; it was run on all the available 12 cores. All the R/Bioconductor packages were run on a single core at a time.

## CONCLUSIONS

In this study, we carried out a practical comparison of the state-of-the-art software packages for detecting differential expression from RNA-seq data at different numbers of replicates to guide the selection of a suitable package. In general, our results demonstrated that there can be large differences between the pipelines and no single method is likely to be optimal under all circumstances.

When the number of replicates is very small (say below 5), the results from statistical testing should always be taken with caution. This is particularly important to keep in mind, as such small numbers of replicates remain a common practice in many RNA-seq studies. In the present comparisons, limma and DESeq seemed to be among the safest choices in such cases in terms of the consistency of the detections with those made from the complete data (Figure 2C). In contrast, edgeR showed a large range of variability, while the non-parametric SAMseq suffered from low power. When the number of samples becomes larger, the choice of the software package becomes less critical, except for NOIseq and Cuffdiff 2 which identified only few genes as the number of replicates increased above five (Figure 2A). However, this depends also on the characteristics of the data, such as heterogeneity. For instance, in the inherently more heterogeneous human data, only DESeq and limma were able to produce low rates of false positives even if the number of samples was increased (Figure 3B). Thus, it would be important for the users to familiarize themselves with the general relationships between and within each sample group using general visualization and quality assessment methods before choosing the analysis tool. For example limma, DESeq and NOIseq are readily coupled with tools for this purpose. When the number of replicates is relatively large (say ∼10 or more), non-parametric methods may be useful especially if the distributional assumptions are violated [8]. In all cases, care needs to be taken when interpreting the results involving lowly expressed genes. To ensure the accuracy of the attained results, it may be informative to run the analyses with more than one software package (Figure 4).

Based on our comparisons, the choice of the normalization method had surprisingly little influence on the outcome of the analysis. Moreover, although we did not conduct a comprehensive study on the effect of all the run parameters, our evaluation suggested that most of the time they do not have a very strong impact on the results (Supplementary Figure S4 and Table S1). Thus, the recommended default parameters are likely to work well under many circumstances. However, more comprehensive evaluation is needed to fully understand the influence of the different run parameters. Notably, during our studies, updated versions of several of the included pipelines were released, which well illustrates the fact that the field is still under heavy development. Our comparisons showed that the differences between the results produced with different package versions can be significant (Supplementary Figure S1).

Overall, despite different study settings, our observations in real data complemented well the observations made in the recent comparison of Soneson and Delorenzi [16] focusing mainly on synthetic data. Taken together, DESeq was often relatively conservative, while edgeR and EBSeq were often too liberal. Limma performed generally well under many circumstances, being also computationally fastest to run. The performance of baySeq was highly variable depending on the data. SAMseq performed well only when the number of replicates was relatively large. Unlike Soneson and Delorenzi [16], we also included into our systematic comparisons NOIseq and Cuffdiff 2, both of which performed surprisingly poorly in the present data sets.

In addition to the performance differences of the pipelines, practical issues also play a major role for many users when making the choice between them. Not all pipelines work on all operating systems and the installation process may be more complicated for some than others. In general, R/Bioconductor based packages are the easiest to install through the common package handling utility. Some pipelines are feasible to use on a single pc, while others require more computing power—limiting the choice of the pipeline for some users. There are also marked differences in the quality and detail of the documentation of the pipelines; some have very comprehensive manuals and user-guides with practical examples (e.g. DEseq, edgeR, limma), whereas others provide more limited instructions and parameter value descriptions (e.g. NOISeq). Many pipelines like edgeR and limma also support more complex experimental designs, in addition to simple two-group setups. In practice, many users are likely to look for a pipeline that in general works reasonably robustly under a wide range of conditions and is relatively simple to use. Therefore, due to the good documentation, fastness and general ease of use and robustness, limma seems a useful tool for these users based on our comparisons.

## SUPPLEMENTARY DATA

Supplementary Data are available online at http://bib.oxfordjournals.org/.

**Key Points**

- A number of software packages have already been developed to identify differentially expressed genes between distinct sample groups in RNA-seq studies. However, there is no clear consensus about the best practices yet.
- The choice of the approach can markedly affect the outcome of the data analysis and no single tool is likely to be optimal under all circumstances.
- The number of replicates and the heterogeneity of the samples should be taken into account when selecting the pipeline.
- In general, limma performed well under many circumstances in the present comparisons, being also computationally fastest to run.

## FUNDING

This work was supported by the Academy of Finland (grant number 127575 to L.L.E.) and JDRF (grant number 2-2013-32 to L.L.E.).

## Supplementary Material

Supplementary Data

## References

[1] Garber M, Grabherr MG, Guttman M, Trapnell C (2011). Computational methods for transcriptome annotation and quantification using RNA-seq. Nat Methods.

[2] Mortazavi A, Williams BA, McCue K (2008). Mapping and quantifying mammalian transcriptomes by RNA-Seq. Nat Methods.

[3] Marioni JC, Mason CE, Mane SM (2008). RNA-seq: an assessment of technical reproducibility and comparison with gene expression arrays. Genome Res.

[4] Robinson MD, McCarthy DJ, Smyth GK (2010). edgeR: a Bioconductor package for differential expression analysis of digital gene expression data. Bioinformatics.

[5] Anders S, Huber W (2010). Differential expression analysis for sequence count data. Genome Biol.

[6] Hardcastle TJ, Kelly KA (2010). BaySeq: empirical Bayesian methods for identifying differential expression in sequence count data. BMC Bioinformatics.

[7] Tarazona S, García-Alcalde F, Dopazo J (2011). Differential expression in RNA-seq: a matter of depth. Genome Res.

[8] Li J, Tibshirani R (2013). Finding consistent patterns: a nonparametric approach for identifying differential expression in RNA-Seq data. Stat Methods Med Res.

[9] Smyth GK (2004). Linear models and empirical bayes methods for assessing differential expression in microarray experiments. Stat Appl Genet Mol Biol.

[10] Trapnell C, Hendrickson DG, Sauvageau M (2013). Differential analysis of gene regulation at transcript resolution with RNA-seq. Nat Biotechnol.

[11] Leng N, Dawson JA, Thomson JA (2013). EBSeq: an empirical Bayes hierarchical model for inference in RNA-seq experiments. Bioinformatics.

[12] Kvam VM, Liu P, Si Y (2012). A comparison of statistical methods for detecting differentially expressed genes from RNA-seq data. Am J Bot.

[13] Bullard JH, Purdom E, Hansen KD, Dudoit S (2010). Evaluation of statistical methods for normalization and differential expression in mRNA-Seq experiments. BMC Bioinformatics.

[14] Trapnell C, Roberts A, Goff L (2012). Differential gene and transcript expression analysis of RNA-seq experiments with TopHat and Cufflinks. Nat Protoc.

[15] Benjamini Y, Hochbergh Y (1995). Controlling the false dicovery rate—a practical and powerful approach to multiple testing. J R Stat Soc B Methodol.

[16] Soneson C, Delorenzi M (2013). A comparison of methods for differential expression analysis of RNA-seq data. BMC Bioinformatics.

[17] Nookaew I, Papini M, Pornputtapong N (2012). A comprehensive comparison of RNA-Seq-based transcriptome analysis from reads to differential gene expression and cross-comparison with microarrays: a case study in *Saccharomyces cerevisiae*. Nucleic Acids Res.

[18] Oshlack A, Robinson MD, Young MD (2010). From RNA-seq reads to differential expression results. Genome Biol.

[19] Dillies MA, Rau A, Aubert J (2013). A comprehensive evaluation of normalization methods for Illumina high-throughput RNA sequencing data analysis. Brief Bioinform.

[20] Smyth GK, Matthew R, Natalie T limma: Linear models for microarray data user’s guide. http://www.bioconductor.org/packages/2.12/bioc/vignettes/limma/inst/doc/usersguide.pdf.

[21] Bottomly D, Walter NA, Hunter JE (2011). Evaluating gene expression in C57BL/6J and DBA/2J mouse striatum using RNA-Seq and microarrays. PLoS One.

[22] Pickrell JK, Marioni JC, Pai AA (2010). Understanding mechanisms underlying human gene expression variation with RNA sequencing. Nature.

[23] Delhomme N, Padioleau I, Furlong EE, Steinmetz LM (2012). easyRNASeq: a bioconductor package for processing RNA-Seq data. Bioinformatics.

[24] Hackett NR, Butler MW, Shaykhiev R (2012). RNA-Seq quantification of the human small airway epithelium transcriptome. BMC Genomics.

[25] Bourgon R, Gentleman R, Huber W (2010). Independent filtering increases detection power for high-throughput experiments. Proc Natl Acad Sci U S A..

[26] Rau A, Gallopin M, Celeux G, Jaffrézic F (2013). Data-based filtering for replicated high-throughput transcriptome sequencing experiments. Bioinformatics.

[27] Zhang M, Yao C, Guo Z (2008). Apparently low reproducibility of true differential expression discoveries in microarray studies. Bioinformatics.

[28] Elo LL, Hiissa J, Tuimala J (2009). Optimized detection of differential expression in global profiling experiments: case studies in clinical transcriptomic and quantitative proteomic datasets. Brief Bioinform.

