# Supplementary material for: Comparison of software packages for detecting differential expression in RNA-seq studies
Source: Brief Bioinform. 2013 Dec 2;16(1):59–70. doi: 10.1093/bib/bbt086 (PMC4293378; doi:10.1093/bib/bbt086)
Supplement: Supplementary Data [file supp_16_1_59__index.html]

Comparison of software packages for detecting differential expression in RNA-seq studies — Comparison of software packages for detecting differential expression in RNA-seq studies — Supplementary Data 

# Comparison of software packages for detecting differential expression in RNA-seq studies

## Supplementary Data

files

**Files in this Data Supplement:**

- Supplementary Data - zip file
