# Supplementary material for: Comparison of software packages for detecting differential expression in RNA-seq studies
Source: Brief Bioinform. 2013 Dec 2;16(1):59–70. doi: 10.1093/bib/bbt086 (PMC4293378; doi:10.1093/bib/bbt086)
Supplement: Supplementary Data [file supp_bbt086_suppl_data.zip › SupplementaryMaterial.pdf]

**Supplementary Material**

# Comparison of software packages for detecting differential expression in RNA-seq studies

Fatemeh Seyednasrollah, Asta Laiho, Laura L. Elo

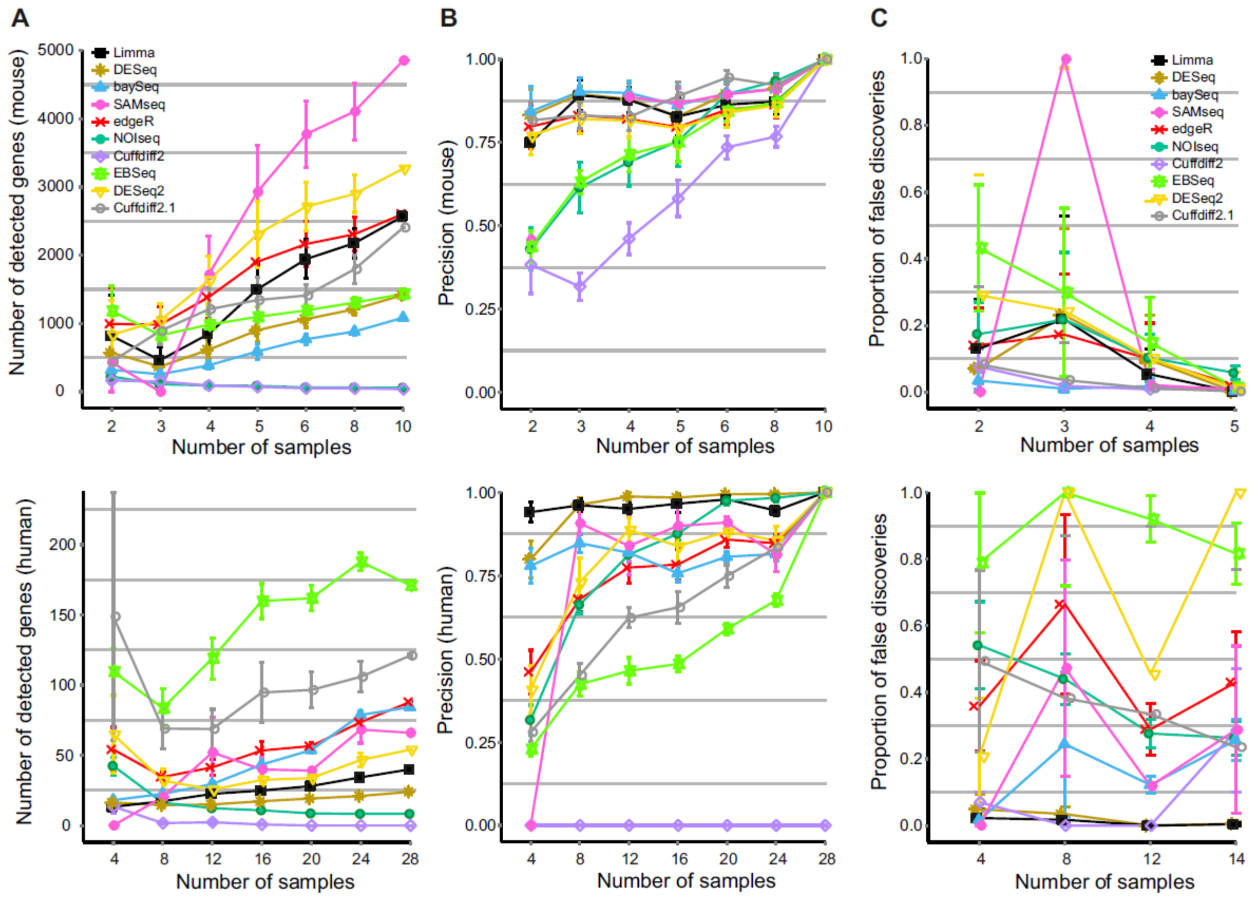


**Figure S1. Performance of DESeq2 and Cuffdiff2.1 compared to the other methods in the mouse and human data (upper and lower panel, respectively). (A)** Number of detections (y-axis) with different numbers of replicates (x-axis) for each software package. The points correspond to averages over ten randomly sampled subsets; the error bars show the standard error of the mean. **(B)** Precision of the detections (y-axis) when increasing the number of replicates (x-axis) in terms of genes identified as differentially expressed genes in the complete data using all the samples available. Only statistically significant genes were considered with each method (see the Methods section for details of the significance thresholds). The points correspond to averages over ten randomly sampled subsets; the error bars show the standard error of the mean. **(C)** False discoveries on the basis of mock comparisons. In each mock comparison, differentially expressed genes were identified between two artificially constructed sample subsets from a single sample group, in which no significant detections are expected. To compare between the different software packages, we divided the number of mock detections with the average number of detections in the actual comparisons with the same number of replicates. Only statistically significant genes were considered with each method (see the Methods section for details of the significance thresholds). The points correspond to averages over ten randomly sampled subsets; the error bars show the standard error of the mean.

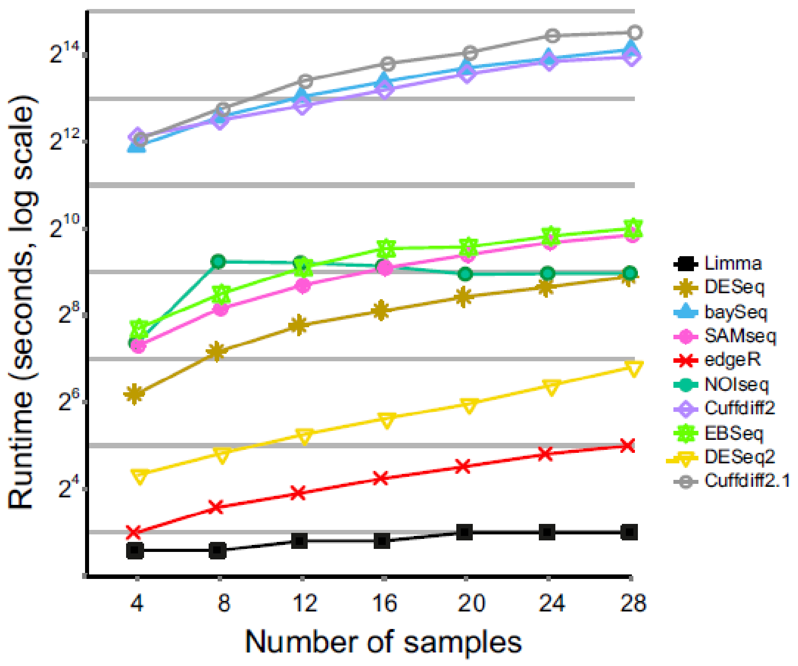


**Figure S2. Runtime of DESeq2 and Cuffdiff2.1 compared to the other methods to identify differentially expressed genes in the human data.** Time in seconds on log scale (y-axis) is shown as a function of the number of replicates (x-axis). The analyses were run on a computer cluster node with two Intel XEON Hexa-Core processors and 96 GB of memory. Cuffdiff2 was run on all the available twelve cores; the R/Bioconductor packages were run on a single core.

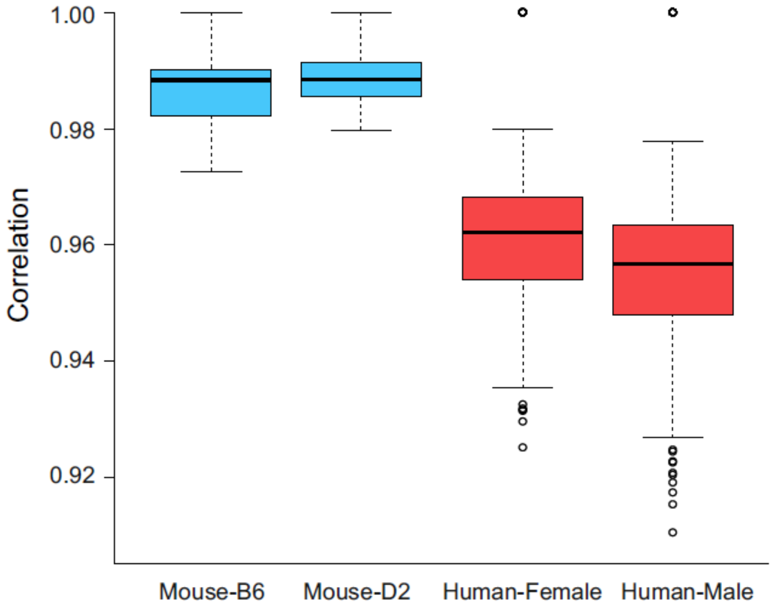


**Figure S3. Homogeneity of the mouse and human datasets.** Spearman correlation values were calculated between replicate samples within each sample group. The boxes show the median and the interquartile range (IQR) of the observed correlations, the whiskers indicate their range and the points correspond to extreme observations with values greater than 1.5 times the IQR. The mouse data showed significantly higher correlations among replicate samples than the human data (Wilcoxon test $p<0.01$), suggesting that they constituted more homogeneous sample groups.

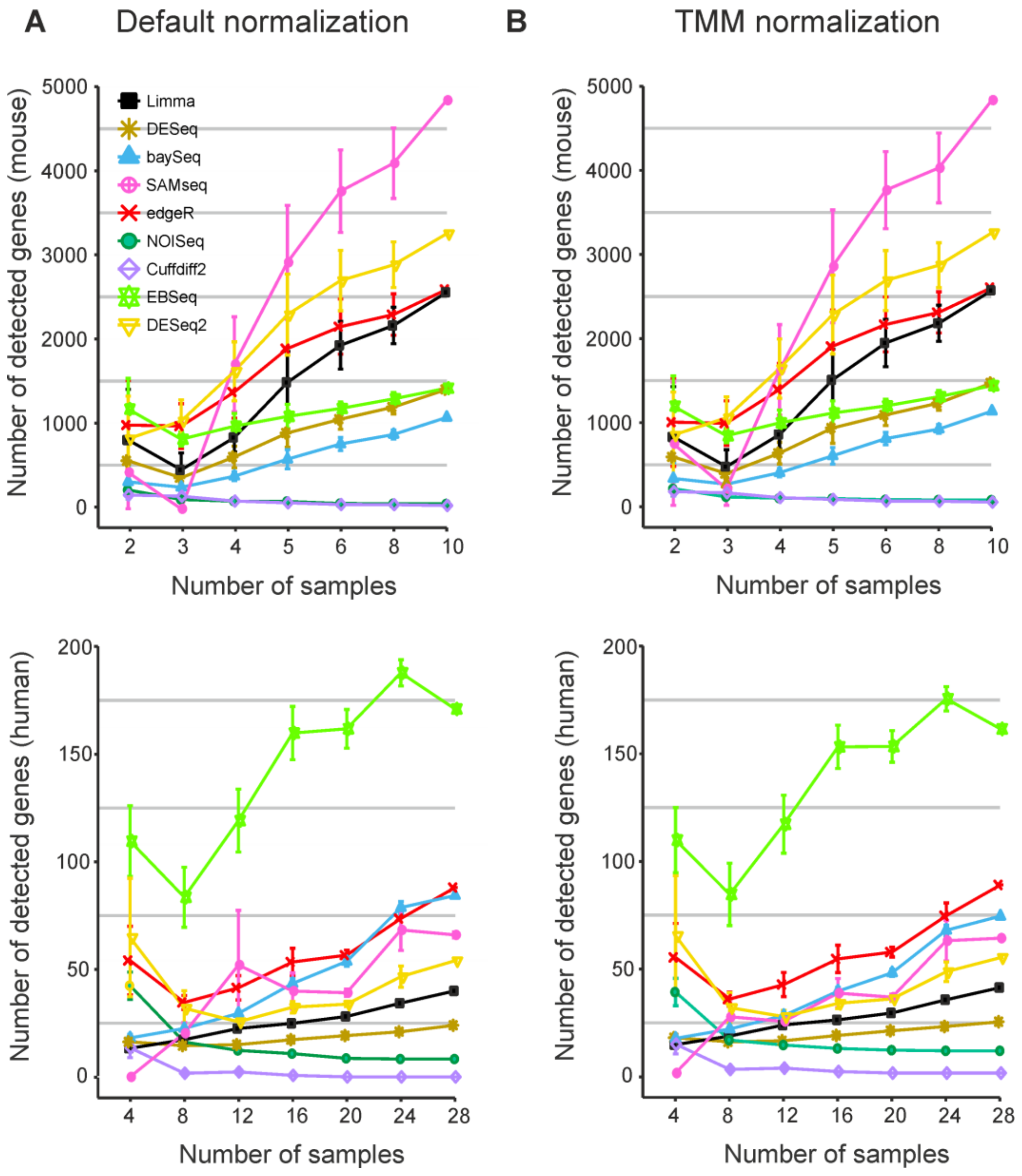


**Figure S4. Comparison between default and TMM normalization.** Differentially expressed genes were detected by eight state-of-the-art software packages in the mouse and human data (upper and lower panel, respectively) using **(A)** their default normalization method or **(B)** the similarly preprocessed data using TMM normalization. For each software package, the number of detections (y-axis) is shown with different numbers of replicates (x-axis). The points correspond to averages over ten randomly sampled subsets; the error bars show the standard error of the mean.

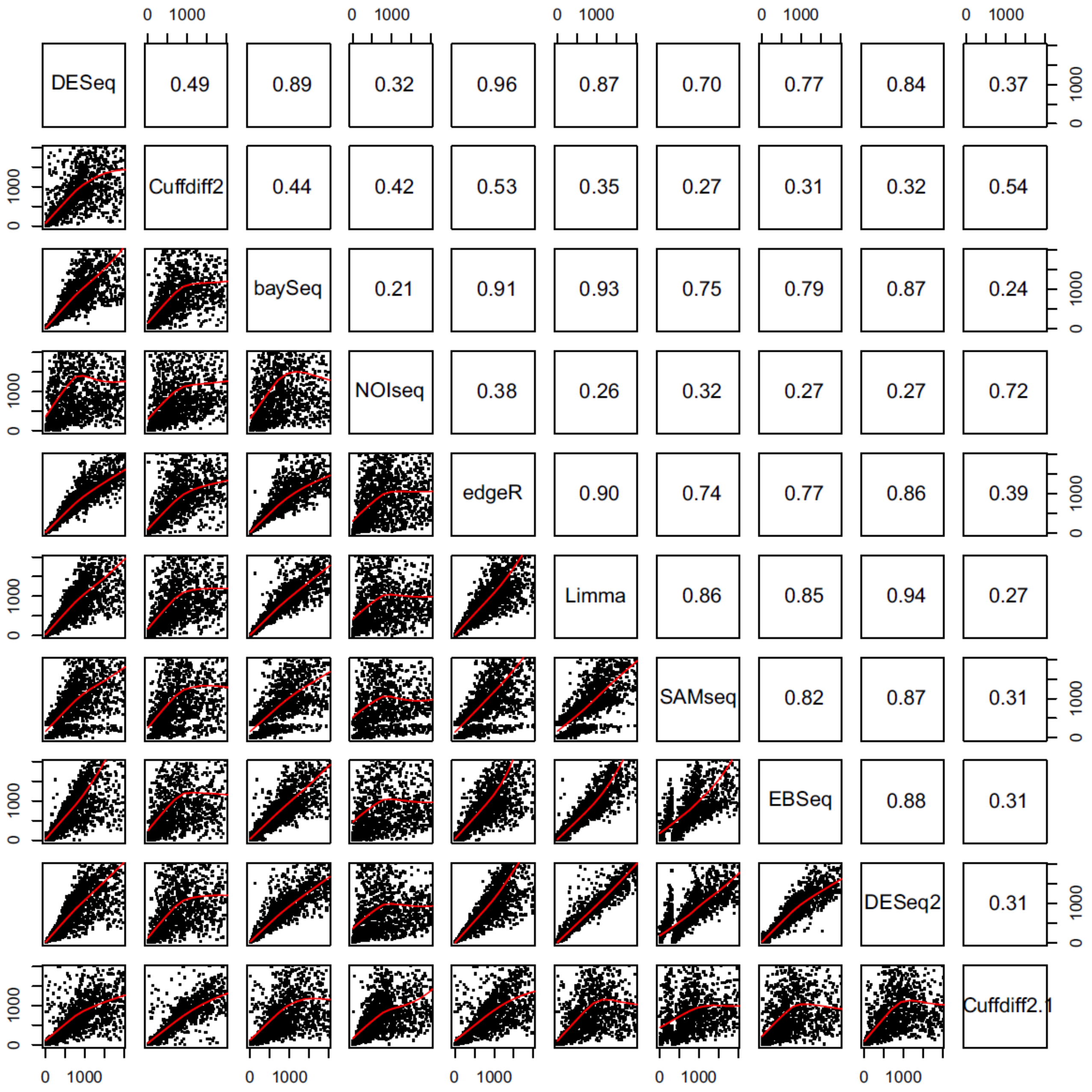


**Figure S5.** Correlations between the gene rankings obtained with state-of-the-art software packages for detecting differential expression in the mouse RNA-seq data. For clarity, to avoid overemphasizing noise, only those 1952 genes that were ranked among the top 1000 most significant genes with any of the methods were considered. The numbers above the diagonal are Spearman correlations of the gene ranks.

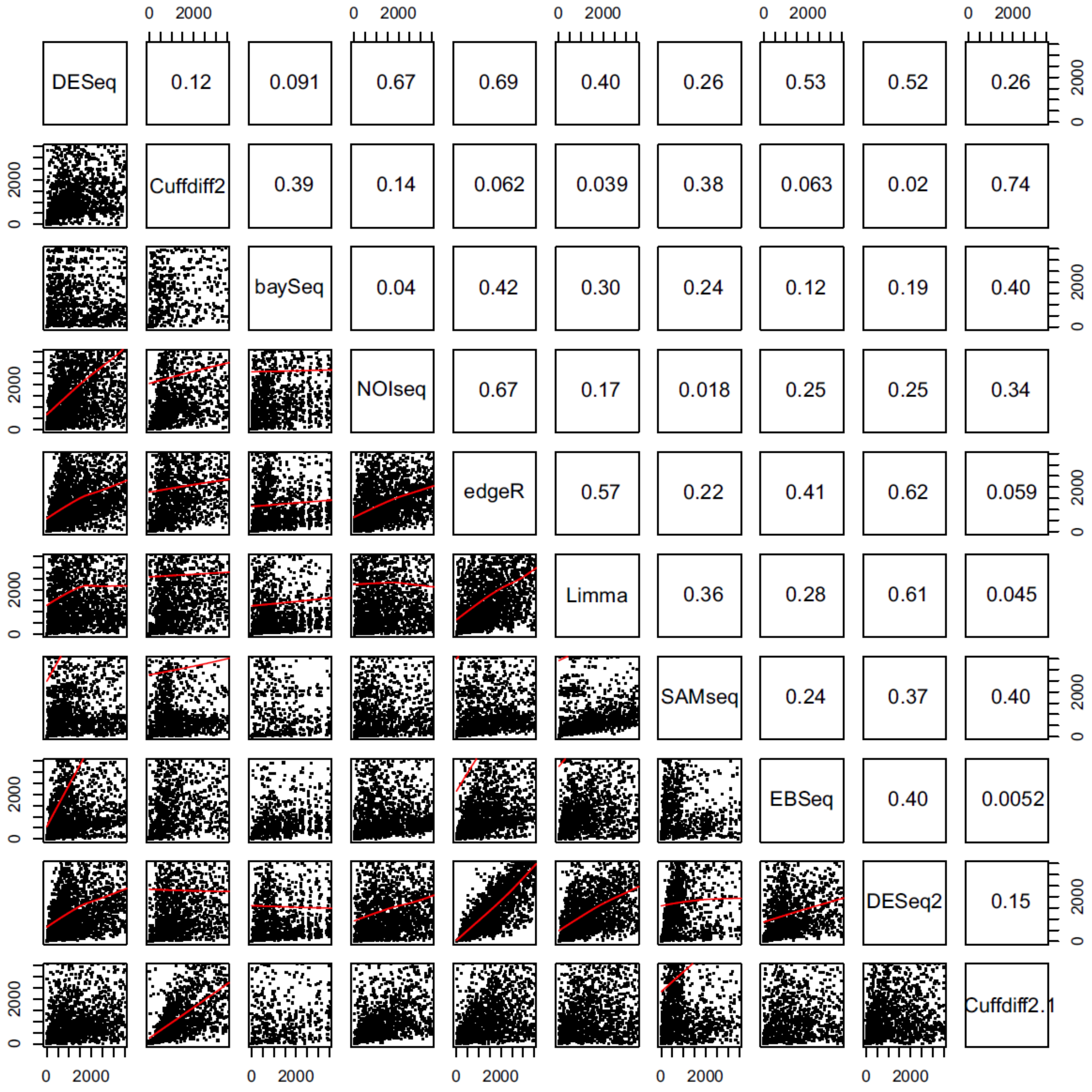


**Figure S6.** Correlations between the gene rankings obtained with state-of-the-art software packages for detecting differential expression in the human RNA-seq data. For clarity, to avoid overemphasizing noise, only those 3443 genes that were ranked among the top 1000 most significant genes with any of the methods were considered. The numbers above the diagonal are Spearman correlations of the gene ranks.

**Table S1.** Number of detections with different versions of Cuffdiff (Cuffdiff2, Cuffdiff2.1 and Cuffdiff1.3.0) and different parameter settings in example datasets with different numbers of replicates.

| | **NUMBER OF DETECTIONS** | | | | | | |
|---|---|---|---|---|---|---|---|
| | **Cuffdiff 2** | | | | **Cuffdiff1.3.0** | **Cuffdiff 2.1** | |
| **Dataset** | **Default parameters** | **Parameter: -u**[a] | **Parameter: -N**[b] | **Parameter: -u and -N** | **Default parameters** | **Default parameters** | **Parameter: -u and -N** |
| Mouse n=2 | 27 | 20 | 27 | 58 | 294 | 1458 | 80 |
| Mouse n=3 | 84 | 74 | 83 | 95 | 81 | 885 | 394 |
| Mouse n=4 | 96 | 82 | 96 | 84 | 84 | 668 | 584 |
| Mouse n=5 | 61 | 50 | 61 | 72 | 56 | 533 | 666 |
| Mouse n=6 | 57 | 48 | 57 | 63 | 40 | 913 | 1246 |
| Mouse n=8 | 59 | 49 | 58 | 62 | 40 | 1245 | 2084 |
| Mouse n=10 | 56 | 39 | 56 | 39 | 33 | 2406 | 2414 |
| Human n=4 | 12 | 22 | 6 | 0 | 13 | 24 | 48 |
| Human n=8 | 5 | 5 | 0 | 0 | 6 | 66 | 40 |
| Human n=12 | 6 | 1 | 1 | 2 | 3 | 64 | 38 |
| Human n=16 | 1 | 1 | 0 | 0 | 2 | 78 | 62 |
| Human n=20 | 0 | 0 | 0 | 0 | 3 | 120 | 99 |
| Human n=24 | 0 | 0 | 0 | 0 | 3 | 133 | 173 |
| Human n=28 | 0 | 0 | 0 | 0 | 3 | 117 | 120 |

[a] Parameter -u enables utilization of an estimation procedure to more accurately weight reads mapping to multiple locations in the genome.

[b] Parameter -N changes the default normalization method (geometric) to upper quartile normalization.

**Table S2.** Number of detections in the real and mock datasets in the mouse data.

| | | Mock data (B6 strain) | | | | Mock data (D2 strain) | | | | Real data |
|---|---|---|---|---|---|---|---|---|---|---|
| **Method** | **Replicates** | **Mean** | **Sem** | **Max** | **Min** | **Mean** | **Sem** | **Max** | **Min** | **Mean** |
| edgeR | 2 | 95 | 87 | 878 | 0 | 185 | 137 | 1399 | 1 | 995 |
| | 3 | 244 | 241 | 2410 | 0 | 95 | 89 | 896 | 0 | 984 |
| | 4 | 55 | 54 | 538 | 0 | 219 | 209 | 2096 | 0 | 1388 |
| | 5 | 16 | 12 | 118 | 0 | 58 | 46 | 470 | 0 | 1905 |
| DESeq | 2 | 17 | 16 | 165 | 0 | 64 | 57 | 577 | 0 | 571 |
| | 3 | 134 | 133 | 1335 | 0 | 37 | 36 | 358 | 0 | 370 |
| | 4 | 1 | 1 | 10 | 0 | 117 | 117 | 1168 | 0 | 617 |
| | 5 | 0 | 0 | 1 | 0 | 1 | 1 | 7 | 0 | 902 |
| Limma | 2 | 173 | 172 | 1718 | 0 | 41 | 27 | 268 | 0 | 819 |
| | 3 | 203 | 203 | 2029 | 0 | 0 | 0 | 0 | 0 | 464 |
| | 4 | 0 | 0 | 0 | 0 | 91 | 91 | 906 | 0 | 848 |
| | 5 | 0 | 0 | 0 | 0 | 0 | 0 | 1 | 0 | 1503 |
| NOIseq | 2 | 41 | 26 | 268 | 1 | 37 | 18 | 188 | 2 | 224 |
| | 3 | 40 | 31 | 321 | 1 | 8 | 4 | 47 | 2 | 111 |
| | 4 | 8 | 6 | 62 | 1 | 11 | 7 | 75 | 2 | 91 |
| | 5 | 6 | 2 | 24 | 1 | 4 | 1 | 14 | 1 | 88 |
| baySeq | 2 | 5 | 4 | 37 | 0 | 17 | 12 | 119 | 0 | 321 |
| | 3 | 1 | 0 | 2 | 0 | 4 | 3 | 32 | 0 | 257 |
| | 4 | 0 | 0 | 0 | 0 | 14 | 12 | 126 | 0 | 388 |
| | 5 | 0 | 0 | 0 | 0 | 4 | 1 | 11 | 0 | 590 |
| Cuffdiff2 | 2 | 20 | 11 | 87 | 0 | 5 | 4 | 42 | 0 | 165 |
| | 3 | 3 | 1 | 8 | 1 | 3 | 1 | 12 | 0 | 150 |
| | 4 | 1 | 0 | 2 | 0 | 1 | 0 | 2 | 0 | 93 |
| | 5 | 1 | 0 | 2 | 0 | 1 | 0 | 4 | 0 | 72 |
| SAMseq | 2 | 0 | 0 | 0 | 0 | 0 | 0 | 0 | 0 | 4335 |
| | 3 | 301 | 336 | 3009 | 0 | 0 | 0 | 0 | 0 | 0 |
| | 4 | 0 | 0 | 0 | 0 | 196 | 438 | 1961 | 0 | 1909 |
| | 5 | 0 | 0 | 0 | 0 | 42 | 40 | 401 | 0 | 2930 |
| EBSeq | 2 | 392 | 192 | 2030 | 20 | 635 | 256 | 2365 | 6 | 1186 |
| | 3 | 310 | 266 | 2700 | 3 | 185 | 146 | 1491 | 4 | 828 |
| | 4 | 105 | 93 | 939 | 0 | 187 | 169 | 1703 | 0 | 982 |
| | 5 | 13 | 6 | 49 | 0 | 19 | 8 | 79 | 0 | 1098 |
| DESeq2 | 2 | 107 | 105 | 1050 | 0 | 378 | 260 | 2619 | 0 | 833 |
| | 3 | 335 | 333 | 3336 | 0 | 174 | 171 | 1711 | 0 | 1045 |
| | 4 | 56 | 55 | 552 | 0 | 278 | 276 | 2761 | 0 | 1634 |
| | 5 | 3 | 2 | 16 | 0 | 50 | 45 | 458 | 0 | 2308 |
| Cuffdiff2.1 | 2 | 5 | 2 | 19 | 0 | 69 | 66 | 662 | 0 | 441 |
| | 3 | 12 | 7 | 70 | 0 | 52 | 38 | 386 | 0 | 891 |
| | 4 | 28 | 28 | 275 | 0 | 1 | 0 | 2 | 0 | 1209 |
| | 5 | 5 | 3 | 30 | 0 | 1 | 0 | 1 | 0 | 1344 |

**Table S3.** Number of detections in the real and mock datasets in the human data.

| | | Mock data (male) | | | | Mock data (female) | | | | Real data |
|---|---|---|---|---|---|---|---|---|---|---|
| **Method** | **Replicates** | **Mean** | **Sem** | **Max** | **Min** | **Mean** | **Sem** | **Max** | **Min** | **Mean** |
| edgeR | 4 | 10 | 3 | 31 | 0 | 29 | 9 | 90 | 1 | 54 |
| | 8 | 28 | 13 | 132 | 2 | 18 | 4 | 39 | 5 | 35 |
| | 12 | 9 | 3 | 36 | 0 | 15 | 3 | 37 | 0 | 41 |
| | 14 | 21 | 8 | 72 | 2 | 25 | 8 | 75 | 0 | 53 |
| DESeq | 4 | 0 | 0 | 3 | 0 | 1 | 1 | 10 | 0 | 16 |
| | 8 | 1 | 0 | 3 | 0 | 0 | 0 | 3 | 0 | 15 |
| | 12 | 0 | 0 | 0 | 0 | 0 | 0 | 0 | 0 | 15 |
| | 14 | 0 | 0 | 1 | 0 | 0 | 0 | 0 | 0 | 17 |
| Limma | 4 | 1 | 0 | 5 | 0 | 0 | 0 | 0 | 0 | 13 |
| | 8 | 0 | 0 | 0 | 0 | 1 | 0 | 3 | 0 | 17 |
| | 12 | 0 | 0 | 0 | 0 | 0 | 0 | 0 | 0 | 23 |
| | 14 | 0 | 0 | 1 | 0 | 0 | 0 | 1 | 0 | 25 |
| NOIseq | 4 | 15 | 3 | 30 | 7 | 24 | 6 | 67 | 6 | 36 |
| | 8 | 7 | 1 | 15 | 0 | 7 | 1 | 11 | 0 | 15 |
| | 12 | 3 | 1 | 6 | 1 | 4 | 1 | 8 | 2 | 12 |
| | 14 | 3 | 1 | 7 | 1 | 3 | 0 | 5 | 2 | 12 |
| baySeq | 4 | 1 | 0 | 3 | 0 | 0 | 0 | 1 | 0 | 18 |
| | 8 | 10 | 7 | 70 | 0 | 1 | 0 | 5 | 0 | 23 |
| | 12 | 3 | 1 | 6 | 0 | 5 | 1 | 8 | 1 | 30 |
| | 14 | 13 | 3 | 30 | 1 | 9 | 2 | 17 | 2 | 43 |
| Cuffdiff2 | 4 | 1 | 0 | 5 | 0 | 1 | 0 | 2 | 0 | 14 |
| | 8 | 0 | 0 | 0 | 0 | 0 | 0 | 0 | 0 | 2 |
| | 12 | 0 | 0 | 0 | 0 | 0 | 0 | 0 | 0 | 2 |
| | 14 | 0 | 0 | 1 | 0 | 0 | 0 | 1 | 0 | 1 |
| SAMseq | 4 | 0 | 0 | 0 | 0 | 0 | 0 | 0 | 0 | 0 |
| | 8 | 5 | 5 | 44 | 0 | 17 | 9 | 73 | 0 | 20 |
| | 12 | 21 | 21 | 210 | 0 | 1 | 0 | 2 | 0 | 52 |
| | 14 | 24 | 14 | 128 | 0 | 1 | 0 | 2 | 0 | 40 |
| EBSeq | 4 | 71 | 24 | 208 | 8 | 102 | 25 | 256 | 32 | 110 |
| | 8 | 149 | 29 | 315 | 43 | 78 | 8 | 115 | 49 | 84 |
| | 12 | 131 | 10 | 184 | 85 | 88 | 6 | 114 | 56 | 119 |
| | 14 | 165 | 11 | 204 | 109 | 96 | 7 | 144 | 69 | 160 |
| DESeq2 | 4 | 8 | 3 | 32 | 0 | 19 | 6 | 59 | 0 | 65 |
| | 8 | 110 | 95 | 955 | 0 | 6 | 4 | 36 | 0 | 32 |
| | 12 | 23 | 22 | 225 | 0 | 1 | 0 | 4 | 0 | 26 |
| | 14 | 35 | 18 | 146 | 0 | 53 | 51 | 516 | 0 | 32 |
| Cuffdiff2.1 | 4 | 91 | 30 | 256 | 0 | 56 | 18 | 207 | 0 | 149 |
| | 8 | 24 | 7 | 60 | 0 | 29 | 8 | 77 | 0 | 69 |
| | 12 | 9 | 4 | 27 | 0 | 37 | 12 | 111 | 0 | 69 |
| | 14 | 11 | 4 | 36 | 0 | 34 | 19 | 197 | 0 | 95 |
