# Supplementary material for: Comparison of software packages for detecting differential expression in RNA-seq studies
Source: Brief Bioinform. 2013 Dec 2;16(1):59–70. doi: 10.1093/bib/bbt086 (PMC4293378; doi:10.1093/bib/bbt086)
Supplement: Supplementary Data [file supp_bbt086_suppl_data.zip › SupplementaryCode.tar!/SupplementaryCode/README.docx]

# In order to use supplementary code files and reproduce the whole study, one can follow the instructions below:

1. The fastq files were downloaded from "http://www.ebi.ac.uk/".

The accession number for mouse data set is: SRP004777.

The accession number for human data set is: SRP001540.

2. Performing quality control using "fastqc"^[1]^ (version: 0.10.1).

3. Performing alignment step using "TopHat2" (version: 2.0.4).

One can use "tophat2.sh" script from this supplementary to do the alignment.

4. To run Cuffdiff2 analyses, it is possible to pass "BAM" files produced by TopHat2 to sample code available in "cuffdiff2.sh" script and run it for different subsample sizes. In this manuscript, we have used Cufflinks (version: 2.0.2-beta).

5. In order to perform analyses using R packages, one should first quantify gene expression levels. In this study, we have used "HTSeq"^[2]^ (version 0.5.3p7) in "htseq.sh" script.

6. The last step is running statistical tests for different R software packages using "script.R" file in this supplementary.

1. ) <http://www.bioinformatics.babraham.ac.uk/projects/fastqc/>
2. ) <http://www-huber.embl.de/users/anders/HTSeq/doc/overview.html>
